# COVID-19 Is Changing Our Understanding of the Neuroscience of Viral Infections: What We Can Do to Prepare for the Future?

**DOI:** 10.3390/neurolint14010007

**Published:** 2022-01-06

**Authors:** Kore Kai Liow, Jason Viereck

**Affiliations:** 1 Department of Medicine (Neurology), John A. Burns School of Medicine, University of Hawaii, Honolulu, HI 96817, USA; jviereck@hawaiineuroscience.com; 2 Neuro COVID Clinic, Brain Research, Innovation & Translation Lab (BRITL), Hawaii Pacific Neuroscience, Honolulu, HI 96817, USA

In the approximately two years since the emergence of COVID-19 (Coronavirus Disease 2019) myriad neurological symptoms have been reported that are seemingly unrelated to each other. Neuropsychiatric syndromes range from stroke and intracranial hemorrhage, to encephalopathy, to anxiety [1]. Management of these neurological manifestations of the virus presents a challenge to clinicians.

COVID-19 can affect the central nervous system both directly and indirectly. The virus may directly gain access to the nervous system via ACE receptors (for example in the nasal mucosa) or as “Trojan Horses” in lymphocytes. As with other systemic infections, especially those causing hypoxia, COVID-19 can indirectly lead to encephalopathy. In addition, the dysfunction of systemic coagulation pathways arising from COVID-19 may trigger stroke. 

As the number of people who have survived infections grows, so too will the number suffering from neurological sequalae. With the virus continuing to evolve, outpatient clinicians can expect to see an increase in cases presenting complex neurological symptoms related to COVID-19.

Knowledge, not only of the expected neurological symptoms but of basic pathophysiology, will help clinicians guide their patients to better outcomes. This would include knowledge of acute, subacute, and chronic syndromes. Research into these issues is ongoing. It is important to encourage continued communication between scientists and clinicians in this rapidly growing field. In the upcoming Special Edition of *Neurology International,* we hope to contribute to this dialogue.
